# Supplementary figures and images for: NKCC1 involvement in the epithelial-to-mesenchymal transition is a prognostic biomarker in gliomas
Source: PeerJ. 2020 Mar 16;8:e8787. doi: 10.7717/peerj.8787 (PMC7081783; doi:10.7717/peerj.8787)

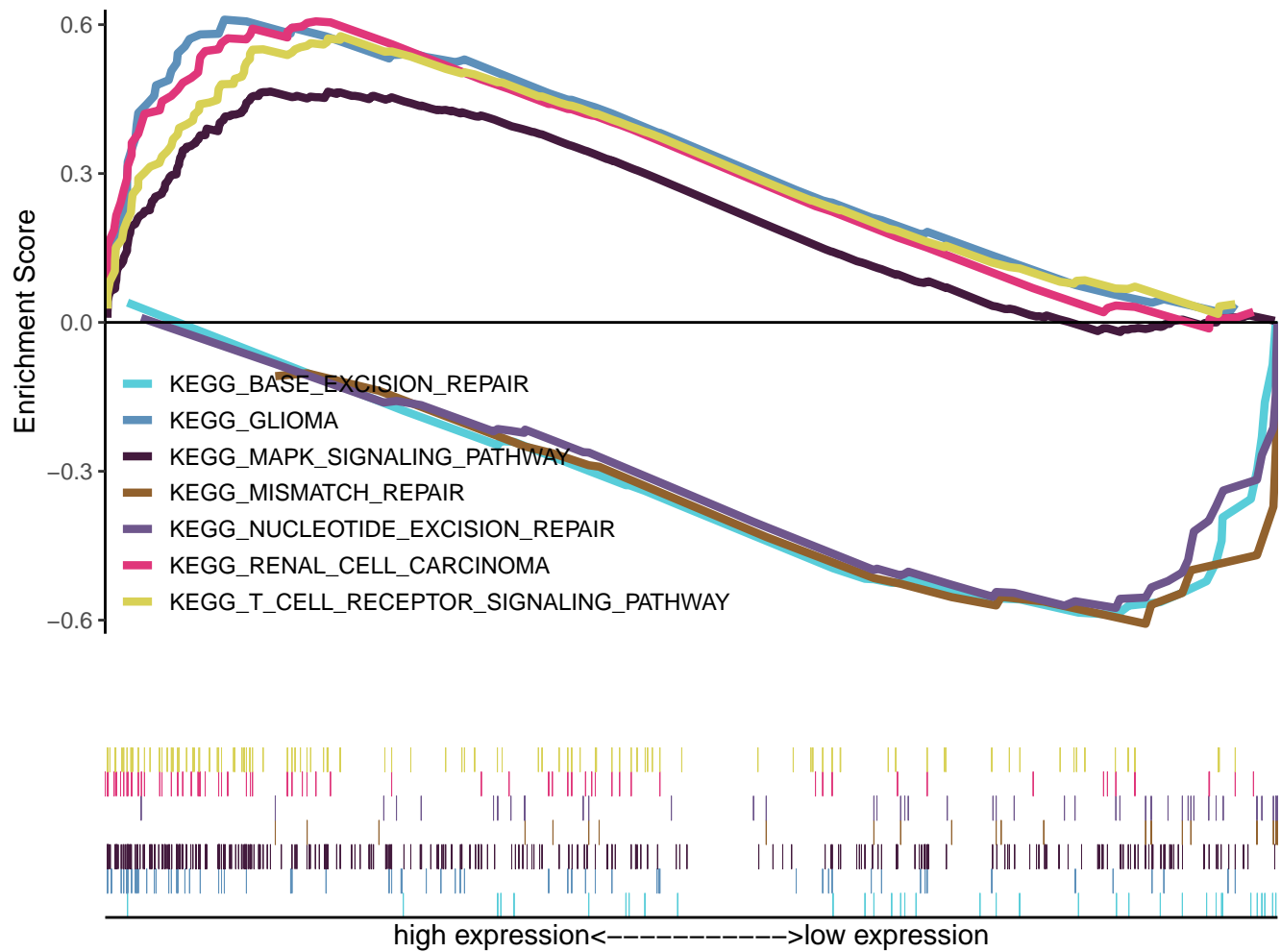

Supplement: Supplemental Information 1 [file peerj-08-8787-s001.zip › GSEA KEGG/multipleGSEA kegg.pdf]

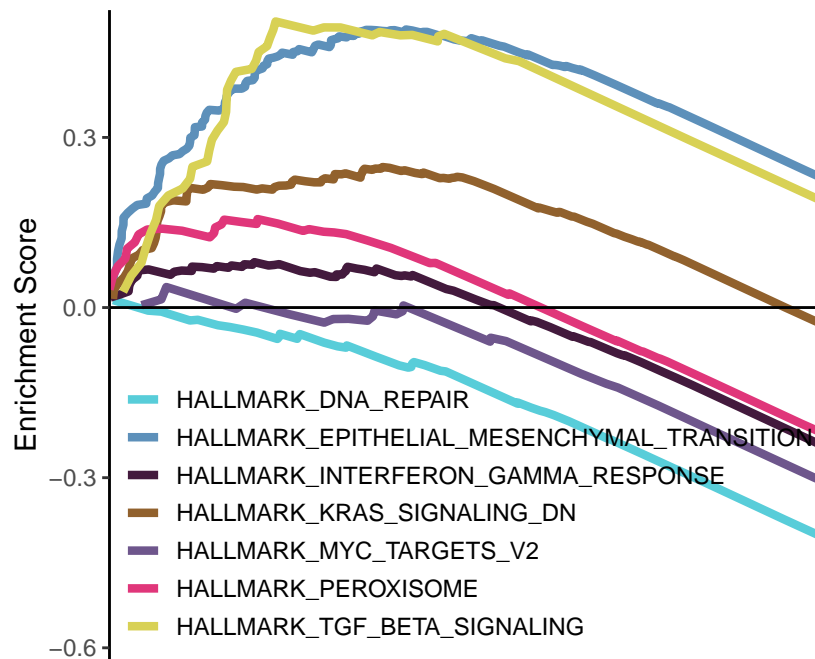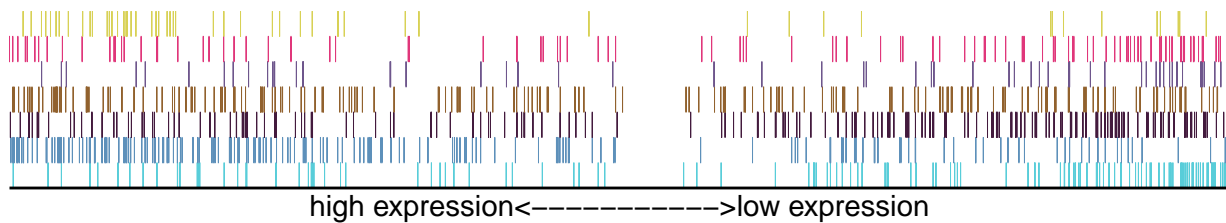

Supplement: Supplemental Information 1 [file peerj-08-8787-s001.zip › HALL GSEA/multipleGSEA hall.pdf]

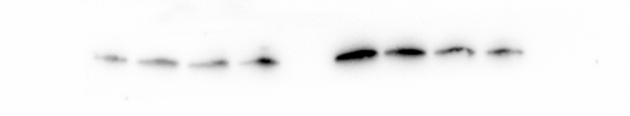

Supplement: Supplemental Information 4 [file peerj-08-8787-s004.zip › MMP-2.png]

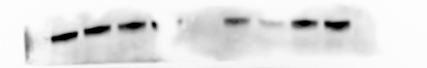

Supplement: Supplemental Information 4 [file peerj-08-8787-s004.zip › MMP-9.png]

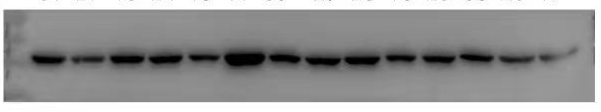

Supplement: Supplemental Information 4 [file peerj-08-8787-s004.zip › NKCC1.png]

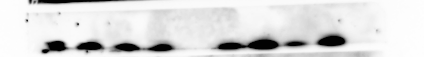

Supplement: Supplemental Information 4 [file peerj-08-8787-s004.zip › β-actin.png]
