# Supplementary material for: NKCC1 involvement in the epithelial-to-mesenchymal transition is a prognostic biomarker in gliomas
Source: PeerJ. 2020 Mar 16;8:e8787. doi: 10.7717/peerj.8787 (PMC7081783; doi:10.7717/peerj.8787)
Supplement: Supplemental Information 3 [file peerj-08-8787-s003.pdf]

Cell Line U251 STR Profile Report

Cell Line Designation: U251

Date Sample Received: Oct 20th, 2017

Report Date: Oct 31th, 2017

Methodology: Twenty short tandem repeat (STR) loci plus the gender determining locus,Amelogenin, were amplified using the commercially available PowerPlex® 21 System from Promega Corporation. The amplified products were processed using the Applied Biosystems 3730xl DNA Analyzer. Data were analyzed using GeneMapper 5.0 software (Applied Biosystems). Appropriate positive and negative controls were run and confirmed for each sample submitted.

Data Interpretation: Cell lines were authenticated using Short Tandem Repeat (STR) analysis as described in 2012 in ANSI Standard (ASN-0002) by the ATCC Standards Development Organization (SDO) and in Capes-Davis et al., Match criteria for human cell line authentication: Where do we draw the line? Int J Cancer.2013;132(11):2510-9.

Genechem

GENECHEM performs STR Profiling following ISO 9001:2008 and ISO/IEC 17025:2005 quality standards.

There are no warranties with respect to the services or results supplied, express or implied, including, without limitation, any implied warranty of merchantability or fitness for a particular purpose. GENECHEM is not liable for any damages or injuries resulting from receipt and/or improper, inappropriate, negligent or other wrongful use of the test results supplied, and/or from misidentification, misrepresentation, or lack of accuracy of those results. Your exclusive remedy against GENECHEM and those supplying materials used in the services for any losses or damage of any kind whatsoever, whether in contract, tort, or otherwise, shall be, at GENECHEM 's option, refund of the fee paid for such service or repeat of the service.

GENE
吉凯基因

TEST RESULTS:

Test Results for Submitted Sample		Reference Database Profile
**Loci**	**Query Profile: U251**	**Database Profile: U251**
**Amelogenin**	X	X
**D13S317**	10 11	10 11
**D16S539**	12	12
**CSF1PO**	11 12	11 12
**TH01**	9.3	9.3
**vWA**	16 18	16 18
**D7S820**	10 12	10 12
**D5S818**	11 12	11 12
**TPOX**	8	8
**D3S1358**	16 17	
**D1S1656**	16 17.3	
**D6S1043**	12	
**Penta E**	7 10	
**D18S51**	13	
**D2S1338**	24	
**Penta D**	12	
**D8S1179**	13 15	
**D12S391**	17 22	
**D19S433**	13 15	
**FGA**	21 25	
**D21S11**	29	
Note: The top nine Loci (8 core STR loci plus Amelogenin) can be made public to verify cell identity. In order to protect the identity of the donor, ***please do not publish*** *the allele calls from all the STR loci tested.*		

Genechem

Percent Match: **100%**

The submitted profile is an exact match for the following human cell line(s) in the reference database (8 core loci plus Amelogenin): **U251**

Explanation of Test Results

Cell lines with ≥80% match are considered to be related; i.e., derived from a common ancestry. Cell lines with between a 55% to 80% match require further profiling for authentication of relatedness. Reference Database Profile was obtained from ExPASy, DSMZ, ATCC or China National Infrastructure of Cell Line Resource STR database.

Applied Biosystems

GeneMapper Software 5

0807

Sample File	Sample Name	Panel	OS	SQ
81_B06.fsa	B06	PowerPlex_21_POP7_Panels_v2.0		

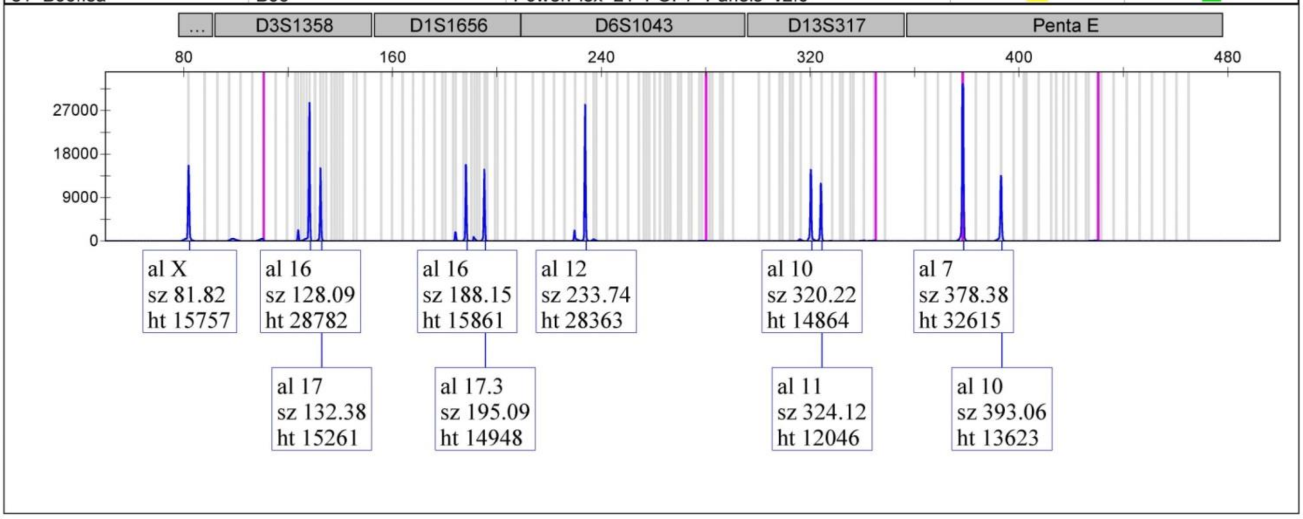

81_B06.fsa	B06	PowerPlex_21_POP7_Panels_v2.0		

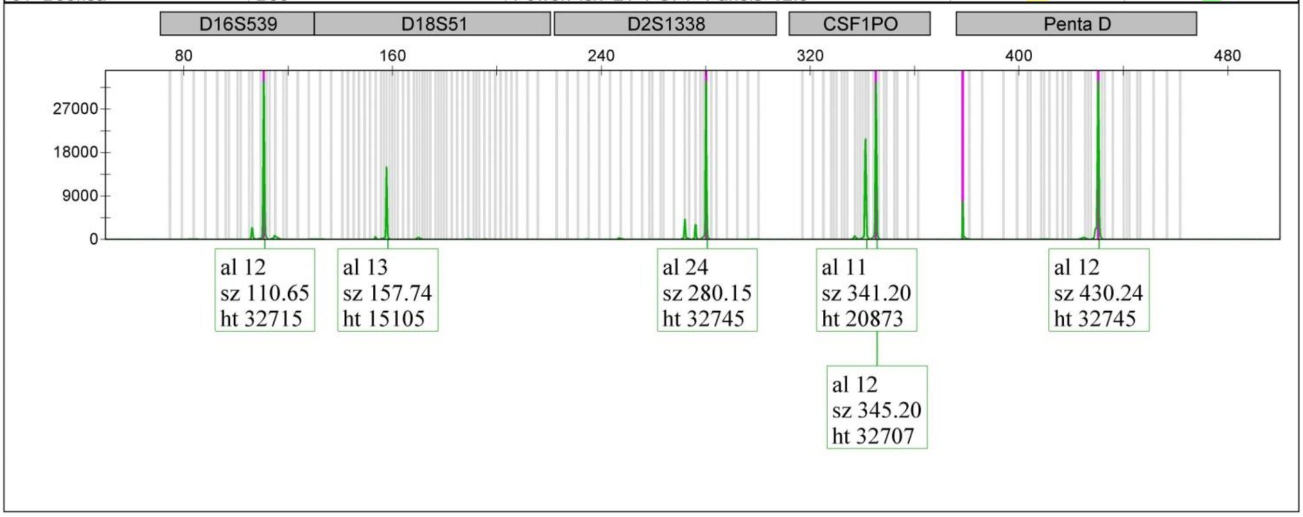

Tue Oct 24,2017 01:14PM, CST Printed by: gm Page 1 of 2

Applied Biosystems

GeneMapper Software 5

0807

Sample File	Sample Name	Panel	OS	SQ
81_B06.fsa	B06	PowerPlex_21_POP7_Panels_v2.0		

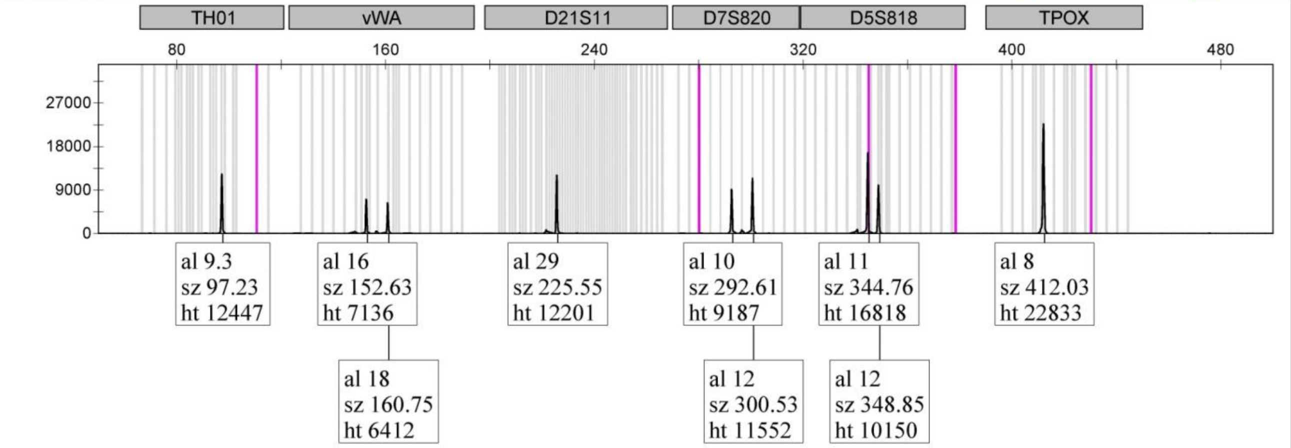

Sample File	Sample Name	Panel	OS	SQ
81_B06.fsa	B06	PowerPlex_21_POP7_Panels_v2.0		

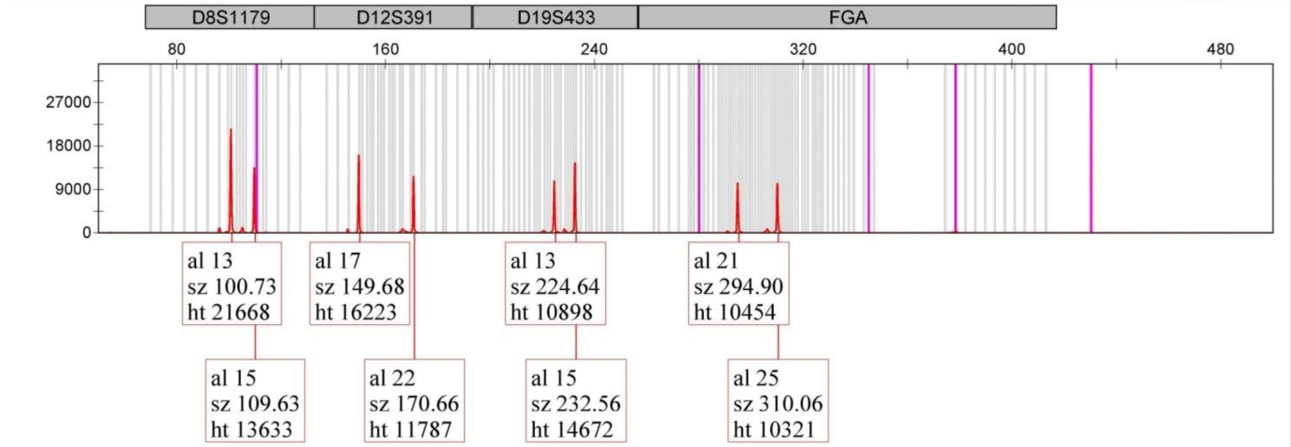

Tue Oct 24,2017 01:14PM, CST   Printed by: gm   Page 2 of 2
